# *In vitro* comparison of various antioxidants and flavonoids from Rooibos as beta cell protectants against lipotoxicity and oxidative stress-induced cell death

**DOI:** 10.1371/journal.pone.0268551

**Published:** 2022-05-17

**Authors:** Céline Moens, Christo J. F. Muller, Luc Bouwens

**Affiliations:** 1 Cell Differentiation Lab, Vrije Universiteit Brussel, Jette, Brussels, Belgium; 2 Biomedical Research and Innovation Platform (BRIP), South African Medical Research Council (MRC), Western Cape, Tygerberg, South Africa; 3 Centre for Cardiometabolic Research in Africa, Division of Medical Physiology, Faculty of Medicine and Health Sciences, Stellenbosch University, Stellenbosch, South Africa; 4 Department of Biochemistry and Microbiology, University of Zululand, KwaDlangezwa, Empangeni, South Africa; Xiangtan University, CHINA

## Abstract

Oxidative stress and lipotoxicity effects on pancreatic β cells play a major role in the pathogenesis of type 2 diabetes (T2D). Flavonoids and antioxidants are under study for their cytoprotective effects and antidiabetic potential. In this study, we aimed to compare the protective effect of the Rooibos components aspalathin, isoorientin, 3-hydroxyphloretin (3-OH) and green Rooibos extract (GRT) itself, and exendin-4 and N-acetylcysteine (NAC) as reference molecules, against lipotoxicity and oxidative stress. The insulin-producing β cell line INS1E was exposed to hydrogen peroxide or streptozotocin (STZ) to induce oxidative stress, and palmitate to induce lipotoxicity. Cell viability was assessed by a MTS cell viability assay. Antioxidant response and antiapoptotic gene expression was performed by qRT-PCR. Glucose transporter 2 (GLUT 2) transporter inhibition was assessed through 2-NBDG uptake. GRT and the flavonoids aspalathin and 3-hydroxyphloretin offered significant protection against oxidative stress and lipotoxicity. GRT downregulated expression of pro-apoptotic genes *Txnip* and *Ddit3*. The flavonoids aspalathin and 3-hydroxyphloretin also downregulated these genes and in addition upregulated expression of antioxidant response genes *Hmox1*, *Nqo1* and *Sod1*. Isoorientin gave no cytoprotection. Cytoprotection by Rooibos components was significantly higher than by NAC or exendin-4. Rooibos components strongly protect INS1E β cells against diabetogenic stress. Cytoprotection was associated with the upregulation of antioxidant response genes of the NRF2/KEAP1 pathway or suppression of the TXN system. The Rooibos molecules offered better protection against these insults than exendin-4 and NAC, making them interesting candidates as β cell cytoprotectants for therapeutic or nutraceutical applications.

## Introduction

Type 2 diabetes (T2D) accounts for about 90% of all diabetes mellitus (DM) cases worldwide [1]. T2D is a metabolic disorder that results in chronic hyperglycaemia due to a relative loss of insulin responsiveness. In a first attempt, β cells will produce more insulin to restore glycaemia homeostasis. However, peripheral tissues become resistant to insulin over time and eventually pancreatic β cells will fail in their attempt to compensate for this increased insulin demand. High circulating glucose (glucotoxicity) together with cellular stress (endoplasmatic reticulum (ER)- and oxidative stress) will cause β cell demise. Lipotoxicity is considered a major risk factor for the development of T2D. Chronic exposure to saturated free fatty acids (FFA), such as palmitate, triggers ER and oxidative stress and results in β cell dysfunction and eventually β cell death [2, 3]. Most current therapies reduce hyperglycaemia by improving insulin sensitivity or insulin replacement. Despite symptomatic alleviation of the disease, preventing β cell loss still remains the most urgent priority for the cure of DM.

In contrast to other cell types, β cells have a low expression of antioxidant enzymes which makes them susceptible for high glucose and free fatty acids induced oxidative stress [4]. Since the pancreas has a weak defense system against oxidative stress, it is suggested that it can be strengthened by external addition of antioxidant molecules. In addition to standard diabetic drugs, antioxidants may represent a promising avenue for the treatment and prevention of T2D [5]. Several studies have already reported a beneficial role of antioxidants in the protection of pancreatic β cells [6].

NAC, the precursor of reduced glutathione (GSH), is an acetylated cysteine residue which acts as a scavenger for reactive oxygen species (ROS) and more particular for hydrogen peroxide [7]. NAC has been attributed antiglucotoxic, antilipotoxic, and antiapoptotic effects and is considered a potent antioxidant [5]. *In vitro*, it was found to decrease oxidative stress in the pancreatic MIN6 β cell line treated with oleic acid [8]. In the RIN-5F β cell line treated with STZ, NAC enhanced insulin secretion, restored the redox homeostasis and protected the cells against STZ-induced apoptosis [7]. NAC prevented energy-depleted cell death and superoxide production in the INS1 β cell line [9]. *In vivo*, NAC prevented hyperglycaemia-induced insulin resistance [10] and preserved glucose tolerance in db/db mice by protecting the β cells [5]. In KK-Ay diabetic mice, NAC improved glucose tolerance and increased insulin sensitivity [11]. NAC also showed to recover the insulin secretion capacity after dexamethasone treatment, reduce intracellular ROS levels and increase cell viability of primary rat islets [12].

Exendin-4, a glucagon-like peptide-1 (GLP-1) receptor agonist, is already used for the treatment of T2D. Exendin-4 showed to reduce palmitate-induced apoptosis in INS1E β cells [2, 13] and both human and murine pancreatic islets [13]. Glucolipotoxicity-induced β cell death was prevented by exendin-4 by improving autophagic flux, reversing lysosomal dysfunction and stimulating autophagy in INS1E β cells and human islets [14]. Exendin-4 has been reported to reduce markers of islet ER stress *in vivo* and enhance survival of INS1 cells and purified rat β cells when challenged with ER stressors thapsigargin and tunicamycin *in vitro* [15].

Our group previously demonstrated that aspalathin, the major polyphenol inherent to the Rooibos plant species, protects insulin-producing INS1E β cells and primary rat islets against glucotoxicity and oxidative stress [16]. This through the upregulation of antioxidant genes via the nuclear factor erythroid 2-related factor 2 (NRF2)-Kelch-like ECH-associated protein 1 (KEAP1)-antioxidant responsive element (ARE) pathway [16]. It has been reported before that the overexpression of antioxidant enzyme genes including *Sod1* and *Hmox1*, protect β cells against ROS toxicity [17]. Oxidized proteins are another consequence of redundant ROS and are reduced by the thioredoxin (TXN) antioxidant system. TXNIP binds and negatively influences the activity of TXN which is important for the β cell’s cellular redox balance [18]. Transcriptional repression of *Txnip* has been proven to be β cell protective and therefore an interesting therapeutic target [16, 18]. Aspalathin is known to have a limited stability and to be prone for oxidative degradation. Isoorientin is the flavone derivative and the major degradation product of aspalathin, besides orientin [19]. It has already been shown that isoorientin from *Gentiana olivieri* plant reduces fasting blood glucose, cholesterol, and triglyceride levels in STZ-induced diabetic rats [20]. Lim et al. demonstrated that isoorientin obtained from the *Sasa borealis* also upregulated NRF2 and increased antioxidant enzymes such as NQO-1 and HMOX-1 [21]. *In silico* and *in vitro* studies suggest the potential of isoorientin as antidiabetic agent [22].

3-Hydroxyphloretin (3-OH PHL), is a dihydrochalcone and aglycone precursor of aspalathin, it has an identical chemical structure as aspalathin but lacks the glucose group. 3-OH PHL is also related to phloretin (PHL), a GLUT-2 inhibitor. It has been proposed that aglycones are more easily taken up by the cell in contrast to their flavonoid glycoside counterparts [23].

GRT is a standardized, pharmaceutical-grade Rooibos extract that contains 12.8% aspalathin, the major polyphenolic compound [2]. It also contains other flavonoids/ polyphenols such as isoorientin, orientin, nothofagin, quercetin, rutin, and PPAG [24]. A detailed overview of the GRT composition can be found in the Material & methods section. *In vivo*, GRT showed to be more effective in an oral glucose tolerance test compared to vildagliptin and lowered plasma glucose levels over 6 h in STZ-induced diabetic Wistar rats, compared to metformin, a first-line antidiabetic drug [25]. In high-fat diet-induced diabetic Vervet monkeys, GRT showed a hypoglycemic effect and reduced low-density lipoprotein levels after two weeks [26].

A comparative study to assess the protective potential of these different antioxidants and flavonoids from Rooibos against different stressors has not been documented so far. The present study was initiated to compare the previously described protective effect of aspalathin [16] relative to other antioxidants/flavonoids from Rooibos, and NAC and exendin-4 as reference molecules, against oxidative stress-induced cell death and lipotoxicity. It was found that Rooibos antioxidants protect INS1E β cells better than exendin-4 and NAC.

## Material and methods

### *In vitro* culture of INS1E β cells

The insulin producing rat insulinoma INS1E β cell line was used for cytotoxicity and gene expression experiments. It was a kind gift of Dr. Claes B. Wollheim at the Centre Médical Universitaire de Genève, Geneva, Switzerland. Passages between 61 and 73 were taken for experiments. The INS1E β cells were grown as previously described [16].

### Compounds

3-(3,4-Dihydroxyphenyl)-1-(3-β-d-glucopyranosyl-2,4,6-trihydroxyphenyl)-1-propanone (aspalathin) and GRT, a pharmaceutical-grade *Aspalathus linearis* extract containing 12.8% aspalathin, were kindly provided by Dr. Christo Müller and Dr. J. Louw of the South African Medical Research Council (Cape Town, South Africa). Aspalathin, a water-soluble dihydrochalcone, was synthesized by High Force Research Ltd (Durham, UK) according to a previously described method, with a purity of ca. 98% based on HPLC (batch SZ1-356-54) [16]. GRT contains several flavonoids (g /100 g extract): 0.423 g PPAG, 1.472 g isoorientin, 1.256 g orientin, 12.783 g aspalathin, 1.041 g quercetin-3-O-robinobioside, 0.339 g vitexin, 0.399 g hyperoside, 0.496 g rutin, 0.298 g isovitexin, 0.572 g isoquercetrin, 1.974 g nothofagin and trace levels of luteolin-7-o-glucoside [24]. 3-Hydroxyphloretin, the water insoluble aglycone of aspalathin, was purchased from TransMIT (Giessen, Germany) with a purity of > 98% based on HPLC and dissolved in 100% ethanol. Isoorientin, the major degradation product of aspalathin, was purchased from Sigma-Aldrich with a purity > 98% HPLC based (Cat. No. I1536) and dissolved in dimethylsulfoxide (DMSO). N-acetyl-l-cysteine (NAC) with > 99% purity (TLC) was purchased from Sigma-Aldrich (Cat. No. A7250). Exendin-4, a GLP-1 receptor agonist, was dissolved in DMSO (Cat. No. ENZ-PRT111; ENZO Life Sciences, Brussels, Belgium).

### *In vitro* cytotoxicity and lipotoxicity treatments

For cytotoxicity experiments INS1E β cells were seeded and treated as previously described [16]. STZ (Sigma-Aldrich) was dissolved at 1 mM in citrate buffer and INS1E β cells were incubated during 1 h. H_2_O_2_ (VWR, PA, USA) was used at a concentration of 125 μM during a 30 min incubation.

For lipotoxicity experiments INS1E β cells were seeded as described above. After 48 h of attachment, cells received compounds at indicated concentrations. After 24 h, culture medium was replaced by 1% FBS containing medium. Compounds were added at indicated concentrations followed by palmitate insult. Briefly, sodium palmitate was dissolved under heat in 90% ethanol (v/v) to a stock concentration of 50 mM. Before insult, palmitate vials were shortly sonicated and heated to 65°C to ensure full solubility. Palmitate was diluted in 0.75% FFA-free bovine serum albumin (BSA; Sigma-Aldrich) containing medium to a final concentration of 0.5 mM before adding to the wells. 90% ethanol (v/v) served as control for palmitate insult. After 18 h, cells were used for analysis.

### MTS cytotoxicity assay

Cell viability/cytotoxicity was measured using the CellTiter 96 Aq_ueous_ One Solution Cell Proliferation Assay (Promega, Madison, USA). This assay quantifies the number of viable cells via a colormetric method. Metabolic active cells are able to reduce tetrazolium compound into a colored formazan product by the presence of NADPH or NADH enzymes. Cell viability percentage was calculated as previously described [16].

### Assessment of apoptotic β cell death by Hoechst-propidium iodide staining

Apoptotic cell death was measured using the DNA binding dyes, Hoechst 33342 (10 μg/mL, Sigma-Aldrich) and propidium iodide (PI, 5 μg/mL, Sigma-Aldrich). After *in vitro* treatments, cells were stained with these dyes for 30 min at 37°C. After staining, cells were washed and fixated with 4% paraformaldehyde for 15 min. After fixation, Dulbecco’s phosphate buffered saline (DPBS) was added to the wells. Cell death images were taken under the Nikon TE2000 Eclipse fluorescence microscope (Nikon, Tokyo, Japan). Quantification of necrotic and apoptotic cells was performed using NIS AR2.30 imaging software (Nikon). Microscopic images (20x magnification) were taken from various regions in the wells to ensure a full representation. Early apoptotic cells were defined as Hoechst positive and PI negative, late apoptotic cells were counted as Hoechst positive and PI positive cells and cells that were only PI positive were counted as necrotic cells. The number of apoptotic and necrotic cells, divided by the total number of cells, represent their percentage. Total percentage of cell death was defined by adding the sum of the percentage necrotic cells to the percentage apoptotic cells. Cell viability was defined by subtracting the percentage of cell death from the total percentage of cells (100%).

### mRNA extraction and Quantitative Reverse Transcription Polymerase Chain Reaction (qRT-PCR)

For gene expression analysis, INS1E β cells were seeded in 6-well plates at a density of 1 million cells per well and incubated for 48 h to guarantee full attachment. After 48 h incubation, cells were treated with the compounds of interest at indicated concentrations for 24 h. After 24 h, cells were harvested for mRNA extraction. mRNA extraction and qRT-PCR were performed as described before [16]. Predesigned SYBRGreen^®^ Kicqstart^®^ primers (Millipore Sigma-Aldrich; primers for the *Sod1* gene) and self-designed DNA oligo primers (Integrated DNA Technologies, IA, USA; all the other primers used) were used. Genes of interest are: *β-actin* (GeneID: 81822), *Nqo-1* (GeneID: 24314), *Hmox1* (GeneID: 24451), *Sod1* (GeneID: 24786, pair 1), *Txnip* (GeneID: 117514), *Blc2* (GeneID: 24224) and *Ddit3* (GeneID: 29467) [16]. Lengths of amplified products were analysed with agarose gel electrophoresis, as well as, dissociation curves to ensure specific amplification of the target gene. Gene expression was calculated relative to the expression level of β-actin housekeeping gene using the delta cycle threshold (Ct).

### 2-NBDG glucose uptake assay

The 2-NBDG glucose uptake assay kit (ab235976, Abcam, Cambridge, UK) employs a fluorescent labeled deoxyglucose analog that can be used for the detection of glucose uptake by cultured cells. Cells were seeded in a 96-well plate with 25 000 cells per well. After 72 h of adherence, cell medium was replaced for 2 h with glucose-free RPMI medium. Afterwards, cells were incubated with indicated compounds for 30 min. Followed by a 15 min incubation with Hoechst 33342 (10 μg/mL, Sigma-Aldrich). 2-NBDG mix was prepared by a 50-fold dilution in glucose-free medium. 2-NBDG mix (50 μL) was added to each well for 10 min at 37°C. Afterwards, the plate was centrifuged for 1 min at 200 *g*. The supernatant was replaced and 50 μL cell-based assay buffer was added. Fluorescence was read at 50 ms integration time using the ImageXpress Pico Automated Cell Imaging System and quantified with the CellReporterXpress Image Acquisition and Analysis Software. All cell average FITC intensities were normalized against nuclei staining and against the control condition.

### Statistical analysis

All statistical analysis were performed using GraphPad Prism 8.0 software (GraphPad Software, San Diego, CA, USA). Differences in cell viability (MTS assay) was determined using a paired one-way analysis of variance (ANOVA) with Sidak’s multiple comparison test. Data of qRT-PCR was analysed by a paired one-tailed Student’s *t*-test. Normality or normality of the residuals (if n were too small) were checked. Normality test indicated Gaussian distribution if not stated otherwise. N indicates independent repeats. All data is expressed as mean ± SEM (Standard Error of the Mean) and analysis was performed at the 0.05 significance level. *p < 0.05, **p < 0.01, ***p < 0.001, ****p < 0.0001, #p < 0.05, ##p < 0.01, ###p < 0.001 and ####p < 0.0001.

## Results

### Cytoprotection against oxidative stress

**Fig 1:**
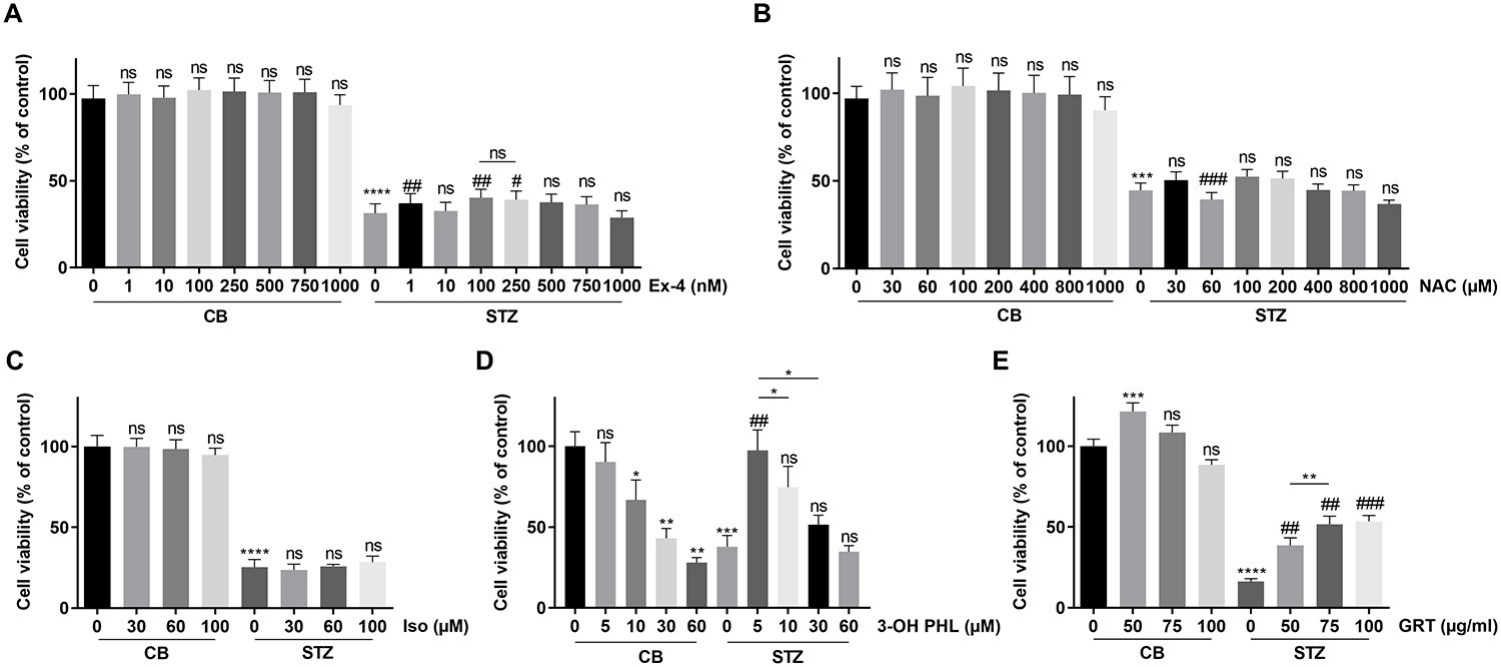
Cytoprotective effect of flavonoid treatment or reference molecules on INS1E β cells after 1.5 mM STZ exposure. INS1E cells were cultured in 96-well plates. Cells were treated with (A) Exendin-4 (Ex-4), (B) N-acetylcystein (NAC), (C) isoorientin (Iso), (D) 3-hydroxyphloretin (3-OH PHL) and (E) GRT, at indicated concentrations 24 h prior to STZ insult and at the time of STZ insult. STZ controls received citrate buffer (CB). After STZ insult, the medium was replaced by fresh medium containing the respective compounds. 18 h after STZ insult, cell viability was measured using MTS assay. Results represent average of independent plates ± SEM. Exendin-4 (n = 15), NAC (n = 9), isoorientin (n = 3), 3-OH PHL (n = 6) and GRT (n = 6). Differences between groups were analysed by paired One-way ANOVA with Sidak’s multiple comparisons test. * above bars indicate comparison against control. # above bars indicate comparison against STZ. * above lines indicate comparison between two treatment groups. Non-significant results are indicated by ns. *p < 0.05, **p < 0.01, ***p < 0.001, ****p < 0.0001, #p < 0.05, ##p < 0.01 and ###p < 0.001.

A first screening for cytoprotective effects against oxidative stress was done with STZ as insult [16]. STZ treatment reduced the viability of INS1E β cells by about 70–80% in the MTS assay (Fig 1). We previously demonstrated the use of the MTS method in assessing cytoprotection [16]. Exendin-4 was able to improve this viability after STZ, with a maximal effect at 100 nM of about 13% (Fig 1A), which we consider a weak cytoprotective effect. NAC, the other reference molecule that was used, was not able to improve the viability in a range of 30–1000 μM after STZ treatment (Fig 1B). We previously showed that the flavonoid aspalathin can partially restore β cell viability after STZ exposure [16], and demonstrate in the present study that its major metabolic degradation product, isoorientin, was not cytoprotective against STZ (Fig 1C). Remarkably, the aglycone of aspalathin, 3-hydroxyphloretin, gave a total protection against STZ-induced cell death at a concentration in the medium of 5 μM. However, at higher concentrations it was cytotoxic to the β cells, even in the absence of STZ treatment (Fig 1D). A pharmaceutical-grade Rooibos extract, GRT, showed a high cytoprotective effect against STZ-induced cell death increasing the viability by 44% (Fig 1E). It should be noted that in the absence of STZ, GRT seemed to increase cell viability over 100% and this effect was also seen in all other experiments with GRT (see below). This must be interpreted not as an effect on cell viability but rather on increased cell proliferation (or metabolic activity) of the INS1E β cells.

**Fig 2:**
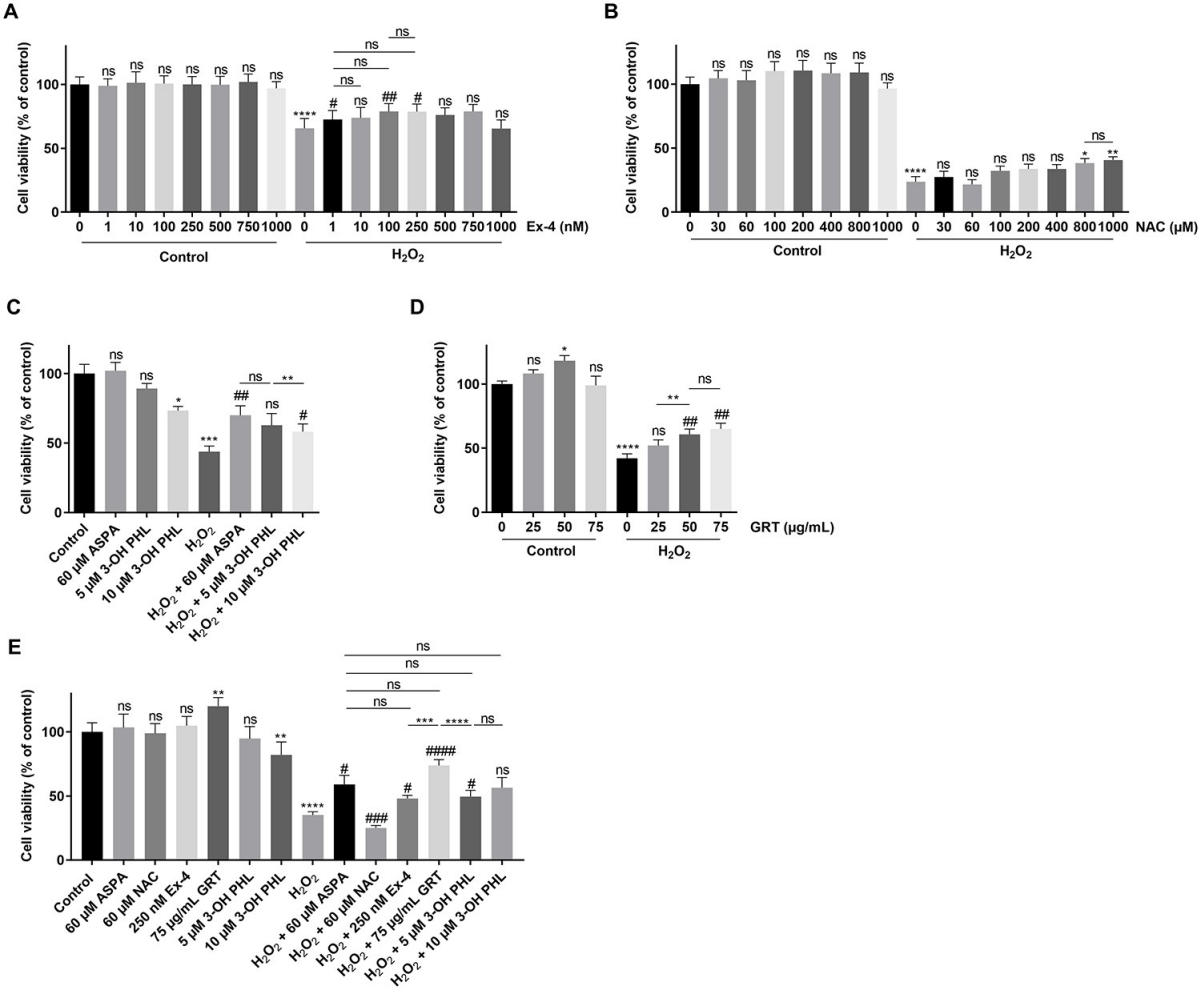
Cytoprotective effect of flavonoid treatment or reference molecules on INS1E β cells after 125 μM H_2_O_2_ exposure. INS1E cells were cultured in 96-well plates. Cells were treated with (A) Exendin-4 (Ex-4), (B) N-acetylcystein (NAC), (C) 3-hydroxyphloretin (3-OH PHL) or aspalathin (ASPA), (D) GRT or (E) all compounds, at indicated concentrations 24 h prior to H_2_O_2_ insult and at the time of H_2_O_2_ insult. After H_2_O_2_ insult, the medium was replaced by fresh medium containing the respective compounds. 18 h after H_2_O_2_ insult, cell viability was measured using MTS assay. Results represent mean of independent plates ± SEM. Exendin-4 (n = 9), NAC (n = 10), 3-OH PHL and ASPA (n = 6), GRT (n = 6) and all compounds (n = 9). Differences between groups were analysed by paired One-way ANOVA with Sidak’s multiple comparisons test. * above bars indicate comparison against control. # above bars indicate comparison against H_2_O_2_. * above lines indicate comparison between two treatment groups. Non-significant results are indicated by ns. *p < 0.05, **p < 0.01, ***p < 0.001, ****p < 0.0001, #p < 0.05, ##p < 0.01, ###p < 0.001 and ####p < 0.0001.

Next, we further examined the compounds that were found to protect against STZ with another type of oxidative stressor, namely H_2_O_2_. Treatment with 125 μM H_2_O_2_ reduced β cell viability by about 60% on average (Fig 2). Reference molecule exendin-4 increased viability by about 38% compared to H_2_O_2_ alone (Fig 2A), and NAC by about 22% (Fig 2B). The flavonoid 3-hydroxyphloretin at a concentration of 10 μM gave some cytoprotection (26%) against H_2_O_2_ as a stressor but this concentration was found to be cytotoxic in the absence of the stressor (Fig 2C). We also compared aspalathin in this setting and found that it increased viability by about 47% (Fig 2C) confirming our previous findings [16]. GRT also cytoprotected β cells from H_2_O_2_-damage with an increased viability of about 40% (Fig 2D).

In order to better compare the various compounds and to dampen the effect of experimental variability, we performed experiments wherein the various compounds were included in the same experiment (at their optimal concentration as found above). As seen in Fig 2E, GRT gave similar cytoprotection to aspalathin, followed in turn by exendin-4 and 3-hydroxyphloretin (5 μM) which gave similar effects. This protective effect was also confirmed by a more sensitive Hoechst-PI staining for aspalathin and 3-hydroxyphloretin (S1 Fig).

### Cytoprotection against lipotoxicity

Besides the use of oxidative stressors in the previous section, we next evaluated whether the compounds would protect the INS1E β cells from lipotoxicity.

**Fig 3:**
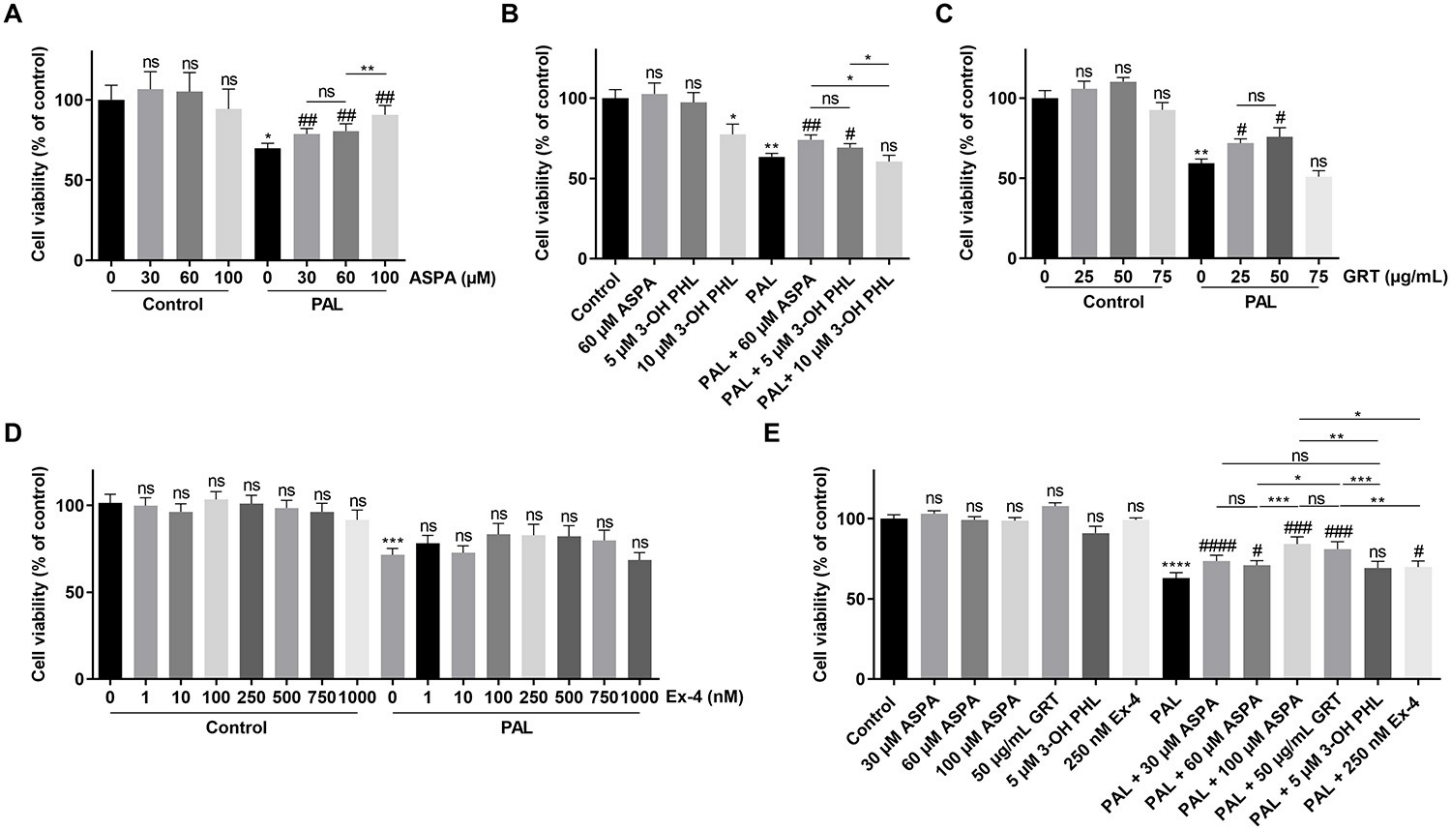
Cytoprotective effect of flavonoid treatment or reference molecule Exendin-4 on INS1E β cells after lipotoxicity. INS1E β cells were cultured in 96-well plates. Cells were treated with (A) Aspalathin (ASPA), (B) 3-hydroxyphloretin (3-OH PHL) or aspalathin (ASPA), (C) GRT, (D) Exendin-4 (Ex-4) or (E) all compounds, at indicated concentrations 24 h prior to 0.5 mM palmitate insult and at the time of palmitate insult. 18 h after palmitate insult, cell viability was measured using MTS assay with. Results represent average of independent plates ± SEM. ASPA (n = 9), 3-OH PHL and ASPA (n = 6), GRT (n = 6), Exendin-4 (n = 8) and all compounds (n = 9). Differences between groups were analysed by paired One-way ANOVA with Sidak’s multiple comparisons test. * above bars indicate comparison against control. # above bars indicate comparison against STZ. * above lines indicate comparison between two treatment groups. Non-significant results are indicated by ns. *p < 0.05, **p < 0.01, ****p < 0.0001, #p < 0.05, ##p < 0.01, ###p < 0.001 and ####p < 0.0001.

INS1E β cells showed a decrease of ~ 30% cell viability when treated with 0.5 mM palmitate in the MTS assay. Aspalathin was also found to significantly increase cell viability after lipotoxic injury and this effect was dose-dependent (Fig 3A). The biggest rescue effect was noticed with 100 μM aspalathin which restored β cell viability with 69%. Also, 5 μM of 3-hydroxyphloretin was found to protect the INS1E β cells against lipotoxicity, although that the observed rescue effect was rather small (16%) and no longer significant with a concentration of 10 μM which was shown to be cytotoxic, as found in the other models mentioned above (Fig 3B). Green Rooibos extract, GRT was also found to protect against palmitate-induced lipotoxicity with a concentration of 25 μg/mL (31%) and 50 μg/mL (40%) (Fig 3C). Reference molecule exendin-4 offered no detectable cytoprotection (Fig 3D).

Lipotoxic rescue effects of the compounds were compared to each other (at their optimal concentration) with the MTS assay, in order to reveal the molecule with the highest protection (Fig 3E). The comparative experiment revealed significant protection against lipotoxicity for all compounds, except for 5 μM 3-hydroxyphloretin which was not significant. GRT (50 μg/mL) was found to protect better against lipotoxicity than 60 μM aspalathin, but showed to be as effective as 100 μM aspalathin (57% vs. 49% respectively). Overall, GRT and aspalathin gave higher β cell viability after palmitate-induced cell death than reference molecule exendin-4 which had only a marginal protective effect in this set of experiments.

### Effects on expression of oxidative stress response genes and pro-apoptotic genes

**Fig 4:**
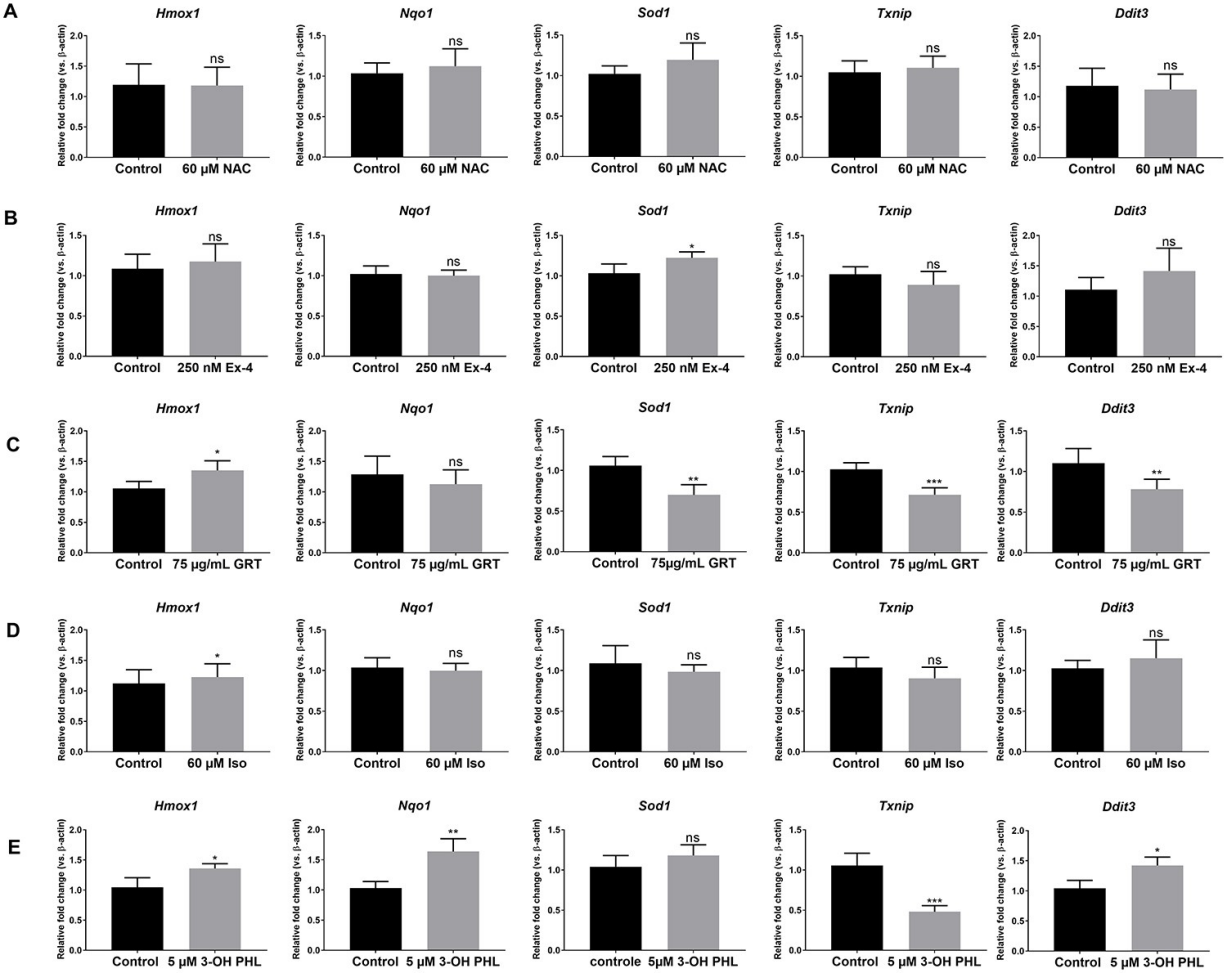
Gene expression analysis of INS1E β cells for antioxidant and apoptotic markers. INS1E β cells were treated with (A) 60 μM NAC, (B) 250 nM exendin-4 (Ex-4), (C) 75 μg/mL GRT, (D) 60 μM isoorientin (Iso) and (E) 5 μM 3-hydroxyphloretin (3-OH PHL). INS1E β cells were treated for 24 h before mRNA extraction. Relative mRNA fold change of antioxidant and apoptotic markers were normalized to *β-actin*. Differences between groups were analysed by paired one-tailed Student’s *t*-test, meanof independent plates ± SEM. NAC (n = 6), Exendin-4 (n = 6), GRT (n = 9), isoorientin (n = 6) and 3-OH PHL (n = 6). *p < 0.05, **p < 0.01 and ***p< 0.001. ns is non-significant.

To understand more about the mechanism by which the compounds offer their protection against oxidative stress, expression of various antioxidant response enzymes was investigated by qRT-PCR. INS1E β cells were analysed for the expression of *Nqo1*, *Hmox1* and *Sod1*. In INS1E β cells, 60 μM NAC (Fig 4A) and 250 nM exendin-4 (Fig 4B) showed no increase in the expression of these antioxidant genes, nor was there increased gene expression of the transcription factor *Nrf2* (S2 Fig). Only a small increase in *Sod1* with 250 nM exendin-4 was noted (Fig 4B). These observations are in line with the absence of a protective effect against STZ-induced oxidative stress observed with NAC and only a small rescue effect noted for NAC and exendin-4 on H_2_O_2_-induced cell death. Additionally, these compounds had no effect on the expression of the oxidative stress-induced pro-apoptotic gene thioredoxin interacting protein (*Txnip*). No effect was found for the gene expression of DNA damage-inducible transcript 3 (*Ddit3*), which is a known pro-apoptotic gene induced under various types of stress (Fig 4A and 4B). GRT (75 μg/mL) on the other hand showed a reduction in *Nrf2* (S2 Fig) and *Sod1* expression, (Fig 4C) in INS1E β cells, which might indicate that its protective potential is not *Nrf2* based, in contrast with was shown previously for aspalathin [16]. However, GRT was found to significantly downregulate pro-apoptotic genes *Txnip* and *Ddit3* (Fig 4C), which suggests protection through an antiapoptotic pathway(s). Exposure of INS1E β cells to aspalathin’s flavone derivative, isoorientin, showed a small increase in *Hmox1* gene expression, but no significant changes in other oxidative or antiapoptotic genes (Fig 4D), which substantiates its lack of protection found in the H_2_O_2_ and STZ model. 3-Hydroxyphloretin treatment markedly upregulated mRNA levels of *Hmox1* and *Nqo1* (Fig 4E). These results are in line with previously published upregulation of antioxidant genes by aspalathin [16] and match the strong cytoprotective effect of its aglycone against H_2_O_2_- and STZ-induced cell death. In addition, 3-hydroxyphloretin significantly repressed pro-apoptotic *Txnip* gene expression, but upregulated *Ddit3* gene expression (Fig 4E).

**Fig 5:**
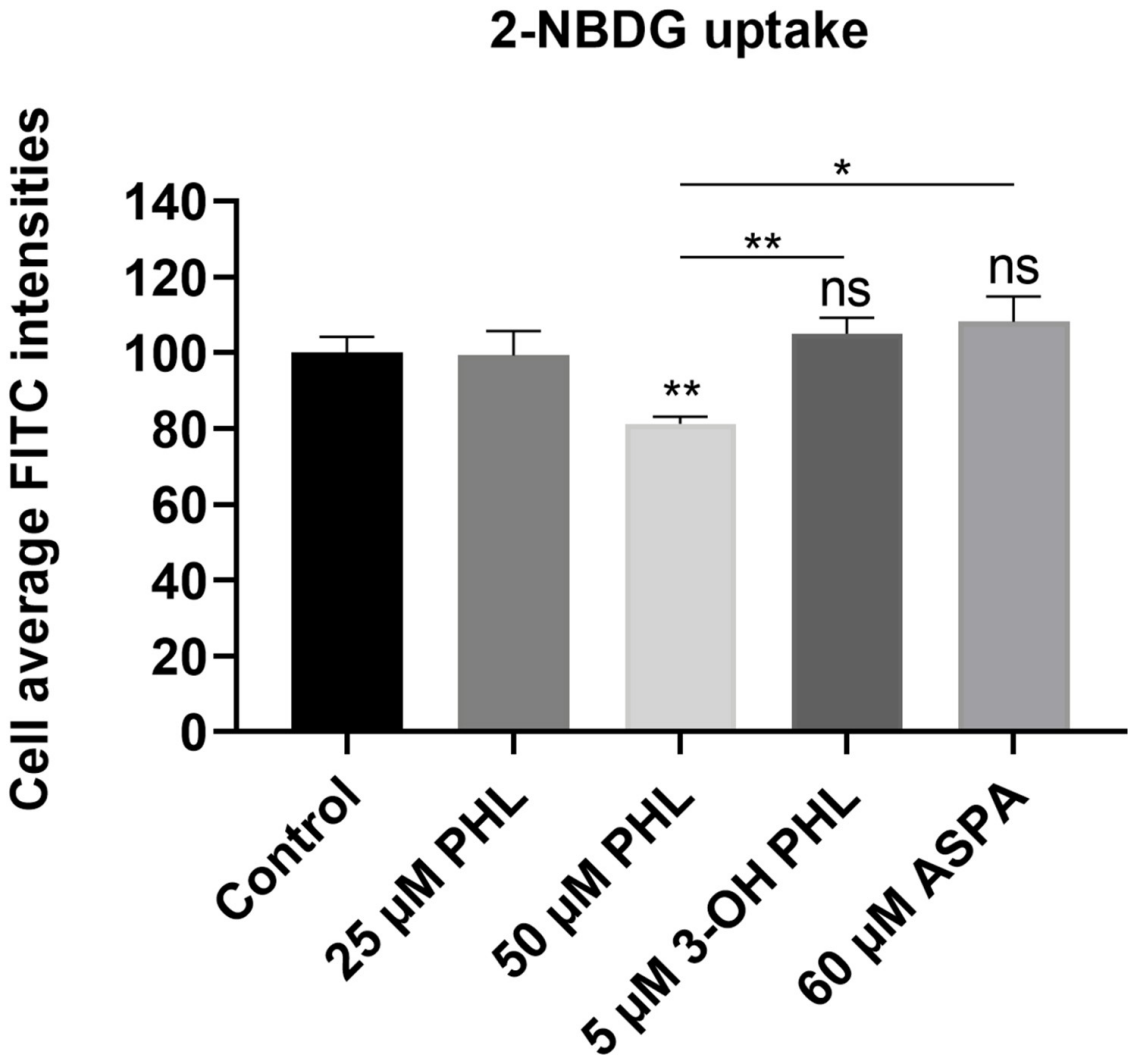
Uptake of fluorescent glucose-analog 2-NBDG. INS1E β cells were exposed to 25 and 50 μM phloretin (PHL; positive control), 5 μM 3-hydroxyphloretin (3-OH PHL) and 60 μM aspalathin (ASPA). Glucose uptake was measured using the ImageXpress Pico Automated Cell Imaging System and quantified with the CellReporterXpress Image Acquisition and Analysis Software. Differences between groups were analysed by paired One-way ANOVA with Sidak’s multiple comparisons test. Results represent average of 8 independent plates ± SEM. Non-significant results are indicated by ns, * p < 0.05 and **p < 0.01.

Since 3-hydroxyphloretin is an analogue of phloretin, a well-known GLUT 2 transporter inhibitor, its potential inhibitor properties on glucose uptake were investigated through 2-NBDG uptake. 2-NBDG is a fluorescent glucose analogue which is taken up by living cells through the GLUT 2 transporter. INS1E β cells were deprived of glucose for 2 h and incubated with 50 μM phloretin, 5 μM 3-hydroxyphloretin and 60 μM aspalathin for 35 min. 3-Hydroxyphloretin administration did not decrease 2-NBDG uptake in INS1E β cells (Fig 5). These observations indicate that 3-hydroxyphloretin does not acutely inhibit the GLUT 2 transporter for glucose uptake. This is also relevant because STZ is taken up by β cells via the GLUT 2 transporter. Aspalathin treatment did not alter 2-NBDG uptake in INS1E β cells, indicating also no GLUT 2 transporter inhibition (Fig 5).

## Discussion

In this study, the ability was examined of several plant-based antioxidant flavonoids, present in the South-African Rooibos plant (*Aspalathus linearis*), to protect pancreatic β cells from cell death induced by oxidative or lipotoxic stress. The ultimate goal is to identify molecules that could be used in the prevention or treatment of diabetes. Other flavonoids have already shown to exert protective effects on β cell survival and function via several mechanisms [27]. These mechanisms include: enhanced antioxidant capacity, improved mitochondrial dysfunction, stimulated insulin secretion, improved insulin resistance, inhibiting gluconeogenesis, decreased glucose absorption in the gut, stimulated prosurvival pathways or inhibited proapoptotic proteins and genes [27]. Thus, flavonoids may be powerful phytochemicals to strengthen the preservation of β cell mass and function [28].

Exposure of INS1E β cells to STZ or H_2_O_2_ are known to induce oxidative stress ultimately leading to β cell death [16, 29, 30]. Oxidative stress is generally caused by an increased production of ROS and free radicals, such as superoxide anion, hydrogen peroxide, hydroxyl radicals and peroxynitrite radicals, causing damage to the DNA, proteins, membrane lipids and is involved in the pathogenesis of diabetes [6]. STZ is a cytotoxin to which β cells are particularly sensitive and can induce DNA fragmentation, decreased ATP synthesis and the formation of hydroxyl radicals and hydrogen peroxide [30]. It is taken up by the β cell via the GLUT 2 transporter in the plasma membrane, as a result of its glucose analogue properties [30].

We previously demonstrated the strong β-cytoprotective effects of the dihydrochalcone flavonoid aspalathin against oxidative stress [16]. Several studies suggest an improved bioactivity of dietary polyphenols when the glycoside group is removed [31, 32]. Therefore, we presently examined 3-hydroxyphloretin, the aglycone of aspalathin, and found that it exhibits cytoprotective effects on β cell survival similar to its glycosylated form. Indeed, 3-hydroxyphloretin at a concentration of 5 μM was able to protect the INS1E β cells against STZ and H_2_O_2_ injuries (Figs 1D and 2C). It was ascertained whether β cell protection against STZ-induced cell death might have occurred due to GLUT 2 transporter inhibition, since STZ is taken up by β cells via the GLUT 2 transporter. Uptake of the glucose analog 2-NBDG was, however, not affected by 3-hydroxyphloretin nor aspalathin (Fig 5). As a control for this, phloretin, a polyphenol found in apple tree leaves which is a well-known GLUT 2 transporter inhibitor, was found to significantly decrease glucose uptake by INS1E β cells (Fig 5). Thus, 3-hydroxyphloretin exerts its protection through another mechanism, most likely the NRF2/KEAP1 antioxidant pathway like previously shown for aspalathin [16]. Indeed, cytoprotection by 3-hydroxyphloretin was associated with an upregulation of the antioxidant genes *Hmox1* and *Nqo1* and a significant repression of the *Txnip* pro-apoptotic gene (Fig 4). Heme oxygenase (*Hmox*) degrades heme to produce carbon monoxide, iron and biliverdin. Its inducible form *Hmox1* is suggested to be increased upon oxidative stress to exert antioxidative and antiapopototic functions in β cells. Enhancement of this gene has shown to alter diabetes development and polymorphisms in the *Hmox1* gene promotor are thought to increase the risk for the development of T2D [33]. Furthermore, upregulation of the *Hmox1* gene was shown to protect rat β cells from oxidative stress [34]. NQO1, another antioxidative enzyme, has been known to protect β cells against oxidative stress, including the stressor STZ [35]. Its primary role is the detoxification of quinones. SOD1 is an important antioxidative enzyme that catalyzes the dismutation of superoxide anion into O_2_ and H_2_O_2_. *Sod1* gene disruption has shown to induce glucose intolerance through β cell dysfunction [36]. It is well reported that these antioxidant genes are regulated by NRF2, a master regulator of antioxidant enzymes [37].

However, at higher concentrations than 5 μM, 3-hydroxyphloretin itself was toxic to the β cells although we don’t know the mechanism of this cytotoxicity yet. This observation makes the glycosylated form, aspalathin, more interesting for potential therapeutic applications since it has no demonstrable toxic effects [16] in contrast with its aglycone form.

Since aspalathin rapidly degrades into one of its major flavone derivative, isoorientin, at physiological pH [19], isoorientin was investigated for its cytoprotective potential. Isoorientin did not offer β cell protection against STZ-induced apoptosis (Fig 1C). In line with these observations, isoorientin did not alter the expression of protective antioxidants genes and did not repress pro-apoptotic genes *Ddit3* and *Txnip* (Fig 4D). It has been described that isoorientin also has less superoxide anion (O_2_^.-^) radical scavenging activity than aspalathin [19, 38]. In general, flavones are less active than their dihydrochalcone counterparts [39].

We aimed to compare the effects of the flavonoids with NAC, a reference antioxidant. Although it has been previously described that NAC offers protection against STZ-induced toxicity in RIN-5F β cells [7], we could not observe protection in the INS1E β cell line (Fig 1B). Only a small protective effect was noticed at concentrations of 800 and 1000 μM (Fig 2B), but no changes in protective antioxidant response genes *Hmox1*, *Nqo1* or *Sod1* were observed (Fig 4A). It has been suggested that the contribution of NAC to the inactivation of H_2_O_2_ and O_2_^.-^ is rather small [40], which may explain the poor cytoprotective outcome in our study. Furthermore, NAC is considered a radical scavenger whereas flavonoids like aspalathin and 3-hydroxyphloretin act as inducers of intracellular antioxidant responses.

We also compared our findings on flavonoids with exendin-4, a well-known antidiabetic drug, that has been shown amongst others to protect mouse β cells and reduce STZ-induced hyperglaecemia [41]. In cardiomyocytes, exendin-4 showed to be protective against H_2_O_2_-induced apoptosis [42]. These results were confirmed in our study, showing protection in a concentration range of 1–250 nM against STZ- and oxidative stress-induced cell death in the INS1E β cell line (Figs 1A and 2A). Exendin-4 showed a small upregulation of the *Sod1* gene, whereas other antioxidant genes like *Hmox1* and *Nqo1* remained unchanged (Fig 4B). It has been reported that exendin-4 attenuates H_2_O_2_-induced oxidative stress in INS1 β cells via translocation of the NRF2 transcription factor to the nucleus, causing the expression of the *Hmox1* gene, but such an effect was not found in our study [43].

Finally, we evaluated the cytoprotective potential of GRT, the green Rooibos extract containing several antioxidants and in particular aspalathin which represents its major polyphenolic compound. It has been demonstrated that GRT prevents hyperglycaemia and oxidative stress in diabetic vervet monkeys [26]. Moreover, it has been reported that green Rooibos tea has relevant anticancer, antimutagenic and anti-inflammatory properties [44]. Here we show, that GRT exerts significant protection against STZ- and H_2_O_2_-induced cell death in the INS1E β cell line (Figs 1E and 2D). It offered significantly better protection than reference molecule exendin-4. However, GRT administration did not alter antioxidant gene expression of *Hmox1* and *Nqo1*, but reduced *Sod1* (Fig 4C). It should be noted that 50 μg/mL GRT contains approximately 14.15 μM aspalathin, which may be insufficient to activate the NRF2-KEAP1 pathway. On the other hand, GRT downregulated the expression of the pro-apoptotic genes *Ddit3* and *Txnip* (Fig 4C).

Not only the NRF2-ARE pathway, but several other antioxidant systems control the β cell’s cellular redox balance, including thioredoxin (TXN) which is a thiol oxidoreductase system [17, 18]. TXN 1 plays an important role in the reduction of oxidized proteins produced by ROS [45]. TXNIP binds and hampers the activity of TXN 1, thus affecting the cellular redox balance [18]. The last few years, TXNIP has emerged as an important regulator of glucose metabolism and pancreatic β cell biology. It has been reported to alter insulin release, glucose production and uptake [18]. Overexpression of *Txnip* has been shown to induce β cell death and β cell dysfunction, whereas *Txnip* knock-out animals where protected from T2D [18]. Hence, *Txnip* has emerged as a promising avenue for therapeutic targeting. It is remarkable that current antidiabetic drugs such as glucagon-like peptide-1 (GLP-1) agonists and metformin have been shown to downregulate *Txnip* expression, which may partially explain their efficacy in the treatment of diabetes mellitus [18]. Previously, we reported aspalathin’s ability to downregulate *Txnip* expression [16]. In this study we show, that also other, related, flavonoid antioxidant molecules such as 3-hydroxyphloretin and GRT are able to downregulate the expression of *Txnip* making them interesting candidates for diabetes treatment. However, the exact antiapoptotic mechanism of GRT in particular deserves to be further investigated.

Another important contributor to β-cell oxidative stress and ER stress is glucolipotoxicity [3]. Elevated FFAs levels are mediators for β-cell dysfunction and death. Aspalathin was also found in this study to be protective for β cells against palmitate-induced lipotoxicity (Fig 3A). This is in line with the findings of Johnson et al. which showed protection with aspalathin from diabetes-induced lipotoxicity in H9c2 cardiomyocytes [46]. Also 3-hydroxyphloretin (Fig 3B) and GRT (Fig 3C) were found to protect the INS1E β cells against lipotoxicity, although the rescue effect was rather small compared to the other stressors. Our comparative study revealed that GRT and aspalathin offered similar levels of cytoprotection and performed much better than exendin-4 (Fig 3E).

## Conclusions

This study reports that an extract from the Rooibos plant *Aspalathus linearis*, and its flavonoid components 3-hydroxyphloretin and aspalathin can protect β cells from oxidative stress and lipotoxicity, partly through the upregulation of antioxidant response genes and partly by the repression of pro-apoptotic genes *Txnip* and *Ddit3*. This study will contribute to the use of Rooibos components for the prevention or treatment of T2D.

## Supporting information

S1 Fig Cytoprotective effect of aspalathin, 3-hydroxyphloretin and reference molecule NAC on INS1E β cells after H_2_O_2_ exposure. INS1E cells were cultivated in 96-well plates. Cells were treated with 60 μM N-acetylcystein (NAC), 60 μM aspalathin (ASPA) or 10 μM 3-hydroxyphloretin (3-OH PHL) 24 h prior to 125 μM H_2_O_2_ insult and at the time of H_2_O_2_ insult. After H_2_O_2_ insult, the medium was replaced with fresh medium containing the respective compounds. 18 h after insult, cell viability was analysed by Hoechst-PI staining. Results represent average of 3 independent plates ± SEM. Differences between groups were analysed by One-way ANOVA with Sidak’s multiple comparisons test. * above bars indicate comparison against control. # above bars indicate comparison against H_2_O_2_. Non-significant results are indicated by ns. ***p < 0.001 and #p < 0.05.
(TIF)

S2 Fig Gene expression analysis of INS1E β cells for *Nrf2* mRNA expression. INS1E β cells were treated with (A) 60 μM NAC, (B) 250 nM Exendin-4 and (C) 75 μg/mL GRT. INS1E β cells were treated for 24 h before mRNA extraction. Relative mRNA fold change of *Nrf2* was normalized to *β-actin*. Differences between groups were analysed by paired one-tailed Student’s *t*-test. Dara are means ± SEM. NAC (n = 6), exendin-4 (n = 6) and GRT (n = 9). **p < 0.01 and ns is non-significant.
(TIF)
