# Supplementary figures and images for: In vitro comparison of various antioxidants and flavonoids from Rooibos as beta cell protectants against lipotoxicity and oxidative stress-induced cell death
Source: PLoS One. 2022 May 17;17(5):e0268551. doi: 10.1371/journal.pone.0268551 (PMC9113568; doi:10.1371/journal.pone.0268551)

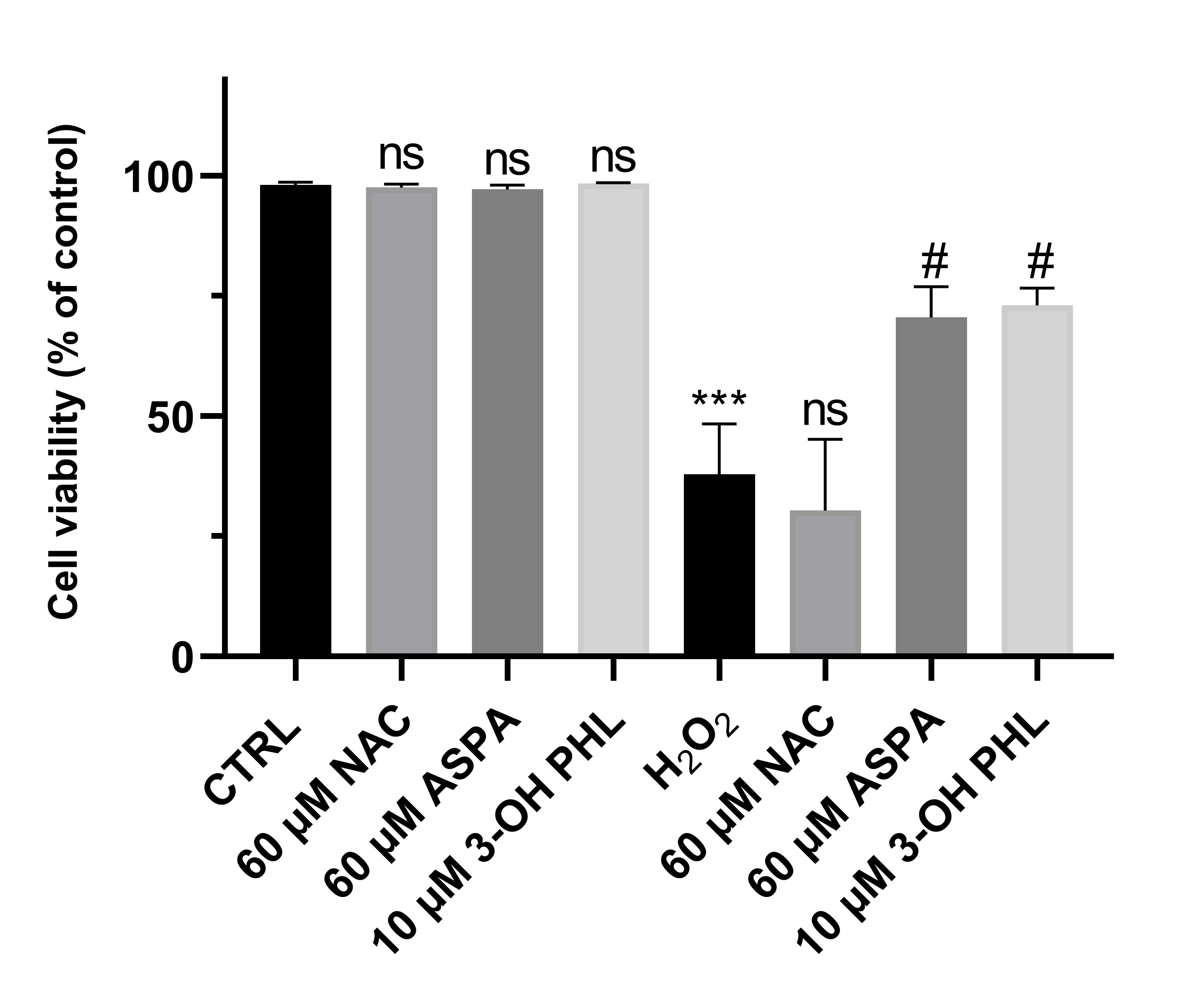

Supplement: S1 Fig — INS1E cells were cultivated in 96-well plates. Cells were treated with 60 μM N-acetylcystein (NAC), 60 μM aspalathin (ASPA) or 10 μM 3-hydroxyphloretin (3-OH PHL) 24 h prior to 125 μM H2O2 insult and at the time of H2O2 insult. After H2O2 insult, the medium was replaced with fresh medium containing the respective compounds. 18 h after insult, cell viability was analysed by Hoechst-PI staining. Results represent average of 3 independent plates ± SEM. Differences between groups were analysed by One-way ANOVA with Sidak’s multiple comparisons test. * above bars indicate comparison against control. # above bars indicate comparison against H2O2. Non-significant results are indicated by ns. ***p < 0.001 and #p < 0.05. (TIF) [file pone.0268551.s001.tif]

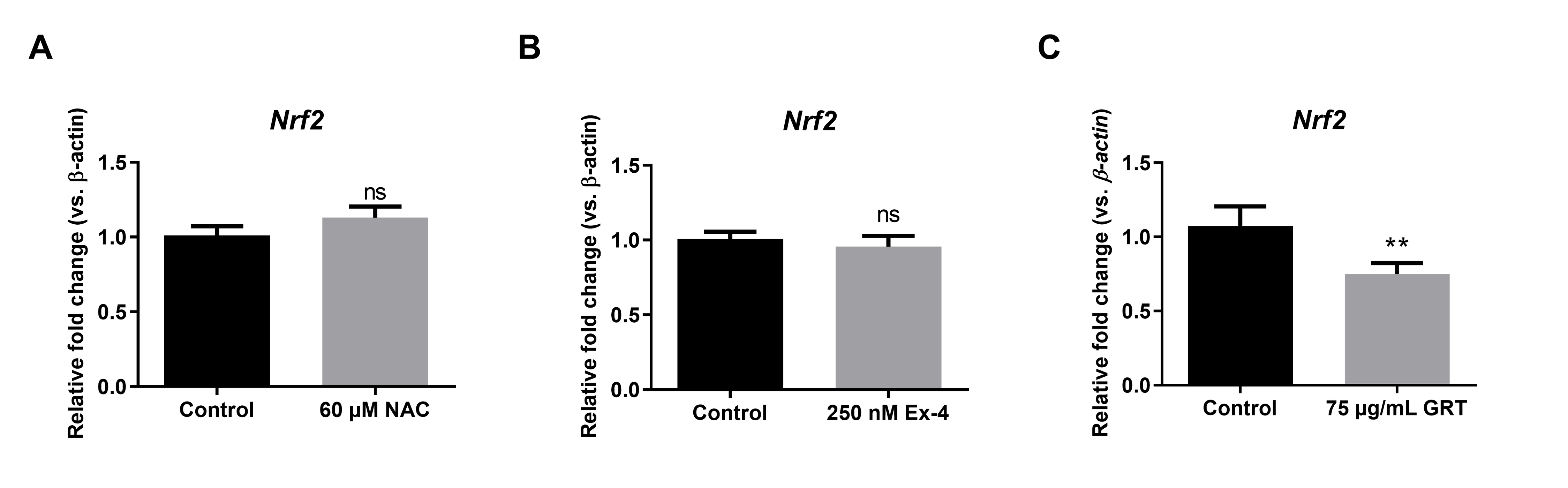

Supplement: S2 Fig — INS1E β cells were treated with (A) 60 μM NAC, (B) 250 nM Exendin-4 and (C) 75 μg/mL GRT. INS1E β cells were treated for 24 h before mRNA extraction. Relative mRNA fold change of Nrf2 was normalized to β-actin. Differences between groups were analysed by paired one-tailed Student’s t-test. Dara are means ± SEM. NAC (n = 6), exendin-4 (n = 6) and GRT (n = 9). **p < 0.01 and ns is non-significant. (TIF) [file pone.0268551.s002.tif]
